# Effect of interleukin (IL)-35 on IL-17 expression and production by human CD4^+^ T cells

**DOI:** 10.7717/peerj.2999

**Published:** 2017-02-15

**Authors:** Kosuke Okada, Takeki Fujimura, Takeshi Kikuchi, Makoto Aino, Yosuke Kamiya, Ario Izawa, Yuki Iwamura, Hisashi Goto, Iichiro Okabe, Eriko Miyake, Yoshiaki Hasegawa, Makio Mogi, Akio Mitani

**Affiliations:** 1 Department of Periodontology, School of Dentistry, Aichi Gakuin University, Nagoya, Aichi, Japan; 2 Department of Microbiology, School of Dentistry, Aichi Gakuin University, Nagoya, Aichi, Japan; 3 Department of Integrative Education of Pharmacy, School of Pharmacy, Aichi Gakuin University, Nagoya, Aichi, Japan

**Keywords:** Interleukin-35, Interleukin-17, Periodontitis, Th17, Retinoic acid receptor-related orphan receptor

## Abstract

**Background:**

Interleukin (IL)-17 produced by mainly T helper 17 (Th17) cells may play an important destructive role in chronic periodontitis (CP). Thus, anti-inflammatory cytokines, such as IL-35, might have a beneficial effect in periodontitis by inhibiting differentiation of Th17 cells. Th17 differentiation is regulated by the retinoic acid receptor-related orphan receptor (ROR) *α* (encoded by *RORA*) and ROR*γ*t (encoded by *RORC*). However, the role of IL-35 in periodontitis is not clear and the effect of IL-35 on the function of Th17 cells is still incompletely understood. Therefore, we investigated the effects of IL-35 on Th17 cells.

**Methods:**

Peripheral blood mononuclear cells (PBMCs) were sampled from three healthy volunteers and three CP patients and were analyzed by flow cytometry for T cell population. Th17 cells differentiated by a cytokine cocktail (recombinant transforming growth factor-*β*, rIL-6, rIL-1*β*, anti-interferon (IFN)-*γ*, anti-IL-2 and anti-IL-4) from PBMCs were cultured with or without rIL-35. *IL17A* (which usually refers to IL-17), *RORA* and *RORC*mRNA expression was analyzed by quantitative polymerase chain reaction, and IL-17A production was determined by enzyme-linked immunosorbent assay.

**Results:**

The proportion of IL-17A^+^CD4^+^ slightly increased in CP patients compared with healthy controls, however, there were no significant differences in the percentage of IL-17A^+^CD4^+^ as well as IFN-*γ*^+^CD4^+^ and Foxp3^+^CD4^+^ T cells between healthy controls and CP patients. *IL17A*, *RORA* and *RORC* mRNA expression was significantly increased in Th17 cells induced by the cytokine cocktail, and the induction was significantly inhibited by addition of rIL-35 (1 ng/mL). IL-17A production in Th17 cells was significantly inhibited by rIL-35 addition (1 ng/mL).

**Discussion:**

The present study suggests that IL-35 could directly suppress IL-17 expression via ROR*α* and ROR*γ*t inhibition and might play an important role in inflammatory diseases such as periodontitis.

## Introduction

Chronic periodontitis (CP) is the most common disease that causes destruction of periodontal tissue (Pihlstrom, Michalowicz & Johnson, 2005). Gram-negative bacterial infection is the main cause for CP, and the host reaction following infection forms a basis for periodontitis. In this regard, T cells, macrophages, epithelium cells and proinflammatory cytokines such as interleukin (IL)-1*β*, IL-6, IL-8 and tumor necrosis factor-*α* (Lundqvist et al., 1994; Sandros et al., 2000) are related to immunoreaction and together function as an immunological barrier for pathogenic bacteria in periodontitis. Many studies have shown the role of cytokines in periodontitis (Genco, 1992; Roberts, McCaffery & Michalek, 1997). The IL-17 cytokine family includes six members (IL-17A–F), and IL-17A is commonly referred to as IL-17. Recent reports have shown that IL-17 is produced by mainly T helper (Th) 17 cells (Abe et al., 2012; Eskan et al., 2012; Gaffen & Hajishengallis, 2008; Takahashi et al., 2014) and interferon (IFN)-*γ* produced by Th1 cells (Teng, 2006) may play an important destructive role in periodontitis. Garlet (2010) reported that Th1 and Th17 cytokines have intimate involvement in the progression of periodontitis. In addition, Th17 cells and IL-17 might be a key inducer for the alveolar bone breakdown in periodontitis (Eskan et al., 2012). Several studies have observed the IL-17 or IFN-*γ* producing T cell population of peripheral blood in CP patients (Chen et al., 2016; Chen et al., 2015; Luo et al., 2014; Saraiva et al., 2013); however, the results are inconclusive as to whether these cells are increased or not.

The role of Th17 cells in periodontitis is still controversial, as Th17 cells have been shown to play a protective and destructive role in infectious diseases and autoimmune diseases (Cua et al., 2003; Kolls & Linden, 2004; Niedbala et al., 2011; Yu et al., 2007). A master regulator gene of Th17 cells is the gene encoding ROR, a member of a nuclear receptor superfamily. ROR has three subtypes, ROR*α*, ROR*β* and ROR*γ* t encoded by *RORA, RORB* and *RORC*, respectively (Ivanov et al., 2006). Th17 differentiation was significantly decreased in a ROR*γ* t-deficient mouse *in vitro* and *in vivo* studies, however the deficiency of ROR*γ* t did not completely suppress Th17 differentiation (Ivanov et al., 2006). This suggests that ROR*γ* t is a critical factor, but an additional transcription factor may also promote Th17 differentiation. Another study reported that ROR*α* regulates Th17 differentiation and both ROR*γ* t and ROR*α* deficiency leads to complete abolishment of Th17 differentiation (Yang et al., 2008).

Anti-inflammatory cytokines from Th2 cells and regulatory T (Treg) cells have been thought to counteract the periodontal disease progression pathways (Garlet, 2010). However, the mechanism for controlling the severity of periodontitis is not clarified. IL-35 is a recently identified anti-inflammatory cytokine heterodimered with Epstein–Barr virus-induced gene 3 (EBI3) and IL-12p35 subunits (Devergne, Birkenbach & Kieff, 1997). IL-35, produced by Treg cells, can downregulate the development of Th17 cell and inhibit autoimmune inflammation (Collison et al., 2012; Collison et al., 2007; Niedbala et al., 2007). IL-35 suppresses IL-17 production by inhibiting differentiation of Th17 cells, and thus plays a protective role in Th17-related diseases (Vignali & Kuchroo, 2012; Whitehead et al., 2012). Accordingly, IL-35 might have a beneficial effect in periodontitis. However, our previous study showed that both IL-35 and IL-17 were significantly upregulated in CP patients (Mitani et al., 2015). Thus, the role of IL-35 in periodontitis is not clear, especially the direct effect of IL-35 on the function of Th17 cells. Therefore, in this preliminary study, we investigated the effect of IL-35 on Th17 cells derived from healthy volunteers, and found a direct inhibitory effect of IL-35 on Th17 cell induction and IL-17 production.

## Materials & Methods

### Participants

CP patients (with at least six teeth with probing pocket depth (PPD) ≥ 5 mm and clinical attachment level (CAL) ≥ 6 mm) and periodontal healthy adult volunteers were recruited from the outpatient population of the Aichi Gakuin University Dental Hospital, Japan from Mar 1 to May 31, 2015. Exclusion criteria were smoking within the past five years, antibiotic therapies during the previous six months, pregnancy and any systemic condition that could affect the progression of periodontitis (e.g., immunological disorders, diabetes and osteoporosis). Healthy volunteers exhibited no signs of clinical periodontal attachment loss with PPD ≤ 3 mm and full-mouth bleeding on probing score <10%. One examiner recorded PPD and CAL as well as demographic data for all participants.

### Ethics statement

All participants signed an informed consent form at the beginning of this study, and the study received approval from the Ethics Committee of Aichi Gakuin University (approval no. 390) in accordance with the World Medical Association Declaration of Helsinki, as revised in 2013.

### CD4^+^T cell isolation from peripheral blood

Peripheral blood was sampled from three healthy volunteers and three CP patients. CD4^+^T cells were purified by the CD4^+^ T cell Isolation Kit (Miltenyi Biotec, Bergisch Gladbach, Germany) and the autoMACS (Miltenyi Biotec), according to the manufacturer’s manual (routine purity; 98%).

### Flow cytometry

For intracellular cytokine staining, the cells were restimulated for 4 h with 50 ng/mL phorbol-12-myristate-13-acetate (PMA) (Sigma-Aldrich, Saint Louis, MO, USA) and 500 ng/mL ionomycin (Sigma-Aldrich) in the presence of Brefeldin A (eBioscience, San Diego, CA, USA), as recommended by the manufacturer. The cells were first stained with APC-eFluor780 conjugated anti-CD4 antibody (Ab), then permeabilized with Perm/Fix solution (eBioscience), and finally stained with PE conjugated anti-IL-17A, eFluor450 conjugated anti-IFN-*γ* and PE-Cy5 conjugated anti-Foxp3 Abs (eBioscience). Data were acquired using a MACSQuant Analyzer 10 (Miltenyi Biotec). Isotype-matched Abs (directly conjugated) were used as controls.

### Th17 cell differentiation and cell culture

Th17 cell differentiation experiments were carried out using samples from three healthy volunteers. We compared Th17 cell differentiation with the cytokine cocktail or rIL-23. CD4^+^T cells (5×10^5^  cells/mL) were cultured for five days in round-bottom 96-well plates with CD3/CD28 T cell expander beads in the presence of cytokine cocktail (recombinant transforming growth factor- *β* (rTGF-*β*) (10 ng/mL), rIL-6 (10 ng/mL), rIL-1 *β* (10 ng/mL), anti–IFN-*γ* (0.5 mg/mL), anti-IL-2 (1 mg/mL) and anti-IL-4 (0.5 mg/mL)) or rIL-23 (40 ng/mL) (Peprotech, Rocky Hill, NJ, USA). After Th17 cell differentiation, Th17 cells were cultured with or without rIL-35 (1 ng/mL) (Peprotech). The cells were collected after 2 h of culture with rIL-35 for polymerase chain reaction (PCR), and the supernatant was collected after 24 h of culture with rIL-35 for enzyme-linked immunosorbent assay (ELISA).

### RNA isolation and quantitative PCR analysis

Total RNA from Th17 cells was immediately isolated using the NucleoSpin RNA II system (Macherey-Nagel, Dueren, Germany) according to the manufacturer’s instructions. The quality of the total RNA was evaluated by measuring the A260/A280nm ratio using a fluorospectrometer (NanoDrop ND-1000; Thermo Scientific, Wilmington, DE, USA). cDNA was synthesized using 13.2 µL of total RNA (40–60 ng/µL), 6 µL of 5× First Strand buffer, 3 µL of DTT (0.1 mM), 6 µL of dNTP Mix (2.5 mM), 0.75 µL of random primers, 1 µL of SuperScript III Reverse Transcriptase (200 units) (Invitrogen, Carlsbad, CA, USA) and 0.5 µL of Ribonuclease Inhibitor (Invitrogen). The reaction was incubated at 37°C for 60 min followed by 5 min at 95°C. To quantify the amount of mRNA, quantitative PCR (qPCR) analyses were performed in a volume of 50 µL containing 2.5 µg cDNA, 28.2 µL TaqMan Universal Master Mix (Applied Biosystems, Foster City, CA, USA), 2.2 µL of each of TaqMan Gene Expression assay (Applied Biosystems) (*IL17A* (Hs99999082_m1), *RORA* (Hs00536545_m1), *RORC* (Hs01076122_m1) and *ACTB* (Hs99999903_s1)) on ABI Prism 7000 Sequence Detection System software version 1.0 (Applied Biosystems) with MicroAmp Optical 96-well Reaction Plate and MicroAmp optical adhesive film (Applied Biosystems). The cycle parameters were as follows: 10 min at 95°C, then 40 cycles of 15 s at 95°C and 1 min at 60°C. Quantification was performed using the ΔΔC_q_ method as previously described (Iwata et al., 2005). Briefly, the fold change between mRNA expression levels was determined as follows: fold change =2^−ΔΔCq^, where ΔΔC_q_ = [(C_q_ target − C_q_*ACTB*(treatedgroup)) − (C_q_target − C_q_*ACTB*(control group))], where “C_q_” denotes the quantification cycle.

### ELISA

IL-17A protein levels in the culture supernatants were measured by human IL-17A ELISA Kits (Diaclone, France) according to the manufacturer’s instructions. The values were recorded at a wavelength of 450 nm and a standard curve was plotted on a linear graph. The values were then calculated from the standard curve.

### Statistical analysis

All data are expressed as mean  ±  SE. Statistical calculations were carried out using GraphPad Prism (Graph Pad Software, San Diego, CA, USA). All data were compared using one-way repeated measures ANOVA followed by the non-parametric Bonferroni multiple comparisons test. Values of *P* < 0.05 were considered significant.

## Results

### T cell population in peripheral blood from CP patients

**Figure 1:**
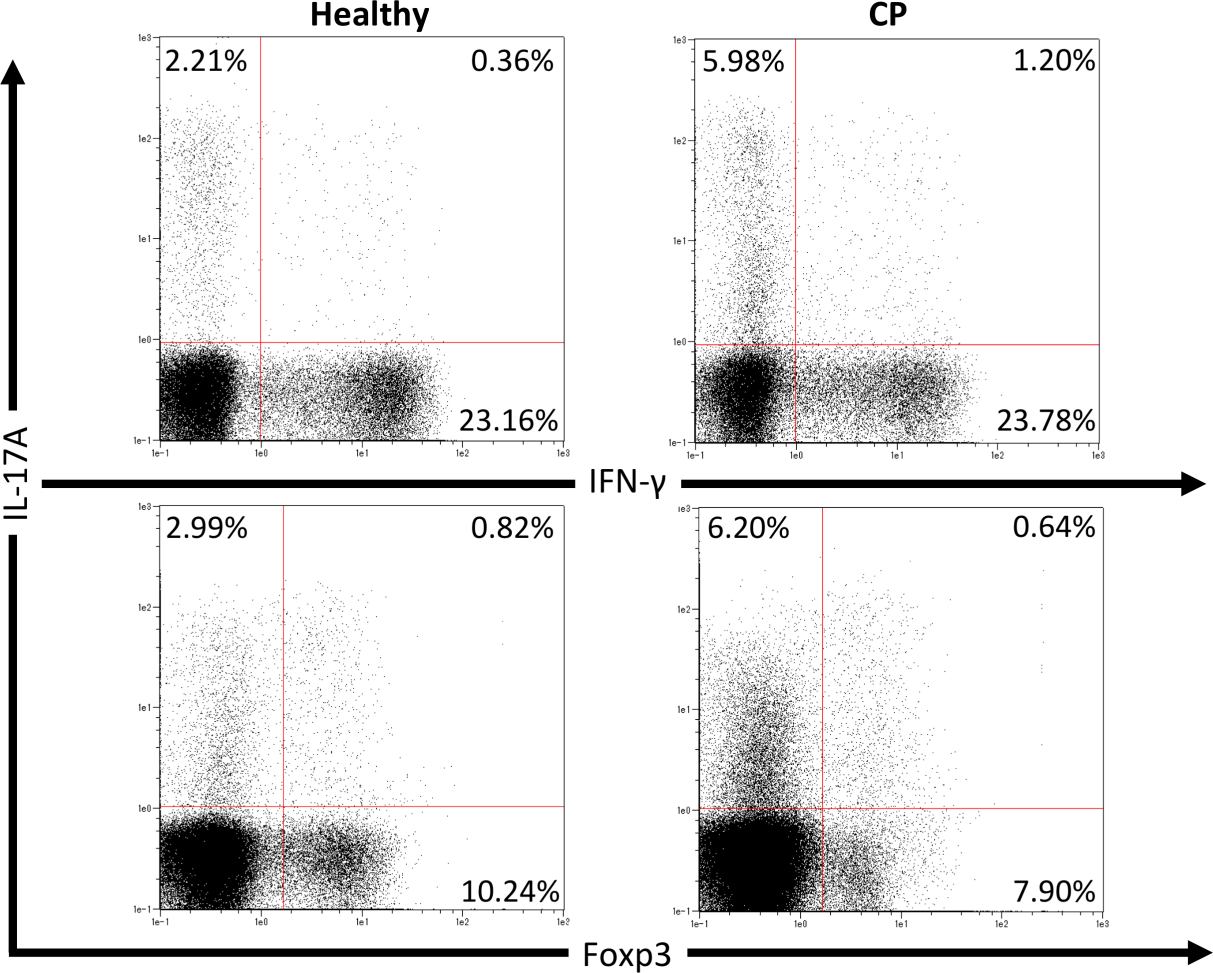
Percentage of IL-17A, IFN-*γ* and Foxp3 expressing CD4^+^ T cells in peripheral blood from healthy volunteers and CP patients. Representative dot plots of IL-17A or IFN-*γ* positive CD4^+^ T cells and IL-17A or Foxp3 positive CD4^+^ T cells in the peripheral blood from healthy volunteers and CP patients. Dot plots are representative of three individuals per group.

We first investigated pathogenic T cell populations in periodontitis by evaluating IL-17A and IFN-*γ* producing CD4^+^T cells in CP patients. We recruited three healthy participants (mean age, 62.0  ±  2.6; one male and two females) and three CP patients (mean age, 52.3  ±  3.7; three females). In healthy participants, the mean PPD was 2.1  ±  0.2 mm and CAL was 2.6  ±  0.2 mm. In CP patients, the mean PPD was 4.0  ±  0.9 mm and CAL was 4.4  ±  1.3 mm. To clarify whether CP affects CD4^+^T cell populations, IL-17A^+^CD4^+^, IFN-*γ*^+^CD4^+^ and Foxp3^+^CD4^+^ T cells in peripheral blood were monitored using flow cytometry. The proportions of IL-17A^+^CD4^+^, IFN-*γ*^+^CD4^+^ and Foxp3^+^CD4^+^ T cells in healthy controls and CP patients were 4.5  ±  1.0%, 22.8  ±  5.5%, 9.4  ±  0.7%, and 7.7  ±  1.9%, 21.3  ±  1.7%, 9.7  ±  0.5%, respectively. One representative dot plot from each group is shown in Fig. 1. No significant differences were observed in any T cell populations between the small sample groups tested.

### IL-35 may directly inhibit IL-17 production in Th17 cells via ROR*α* and ROR*γ*t inhibition

**Figure 2:**
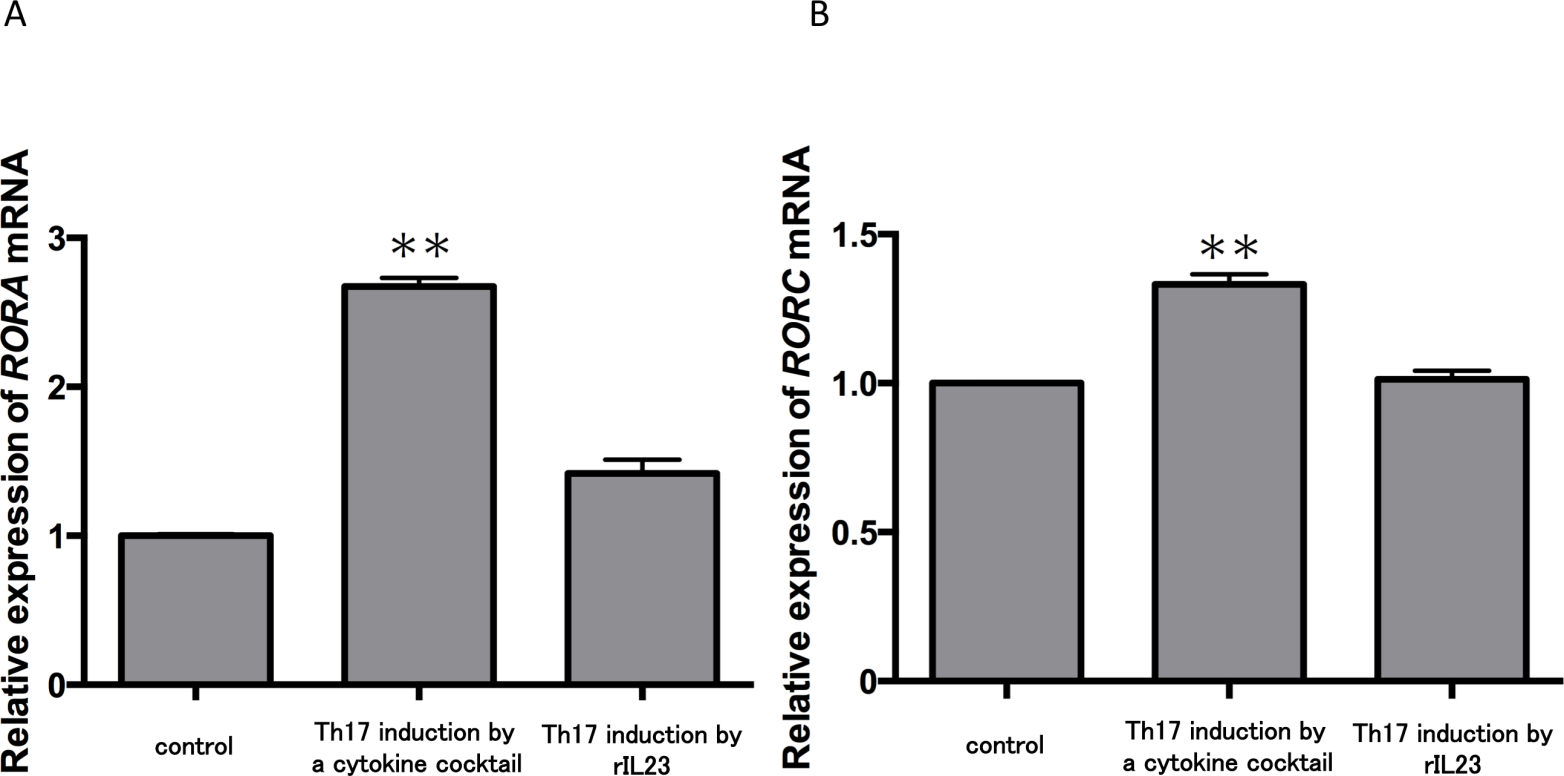
Expression levels of *RORA* and *RORC* mRNA in two Th17 cell differentiation strategies. CD4^+^ T cells in peripheral blood from three healthy volunteers were incubated with a cytokine cocktail or rIL-23 for Th17 cell differentiation. *RORA* (A) and *RORC* (B) mRNA levels were evaluated and normalized to *ACTB*. Values are shown as the mean ± standard error of three independent experiments. ^∗∗^*P* < 0.01 , one-way repeated measures ANOVA followed by the non-parametric Bonferroni multiple comparisons test.

Because no report has shown the direct effect of IL-35 on Th17 cells, we next examined this question. Currently, two major methods are used to induce Th17 cells from CD4^+^ T cells (Langrish et al., 2005; Veldhoen et al., 2009). One is induction by a cytokine cocktail including TGF-*β*, IL-6, IL-1*β*, anti-IFN-*γ* Ab, anti-IL-2 Ab and anti-IL-4 Ab; the second involves addition of rIL-23. We used CD4^+^ T cells in peripheral blood from three healthy volunteers and monitored the expression of master genes for Th17 cells during the two induction strategies. The cytokine cocktail group demonstrated significantly increased expression levels of *RORA* (Fig. 2A) and *RORC* (Fig. 2B) mRNA compared with the control (*P* < 0.01). In contrast, there were no significant changes of *RORA* and *RORC* mRNA expression in the IL-23 treatment group. These results confirmed some reports showing that TGF-*β* and IL-6 but not IL-23 induces Th17 cell differentiation (Mangan et al., 2006; Veldhoen et al., 2006). Thus, we chose the cytokine cocktail induction method for the following experiments.

**Figure 3:**
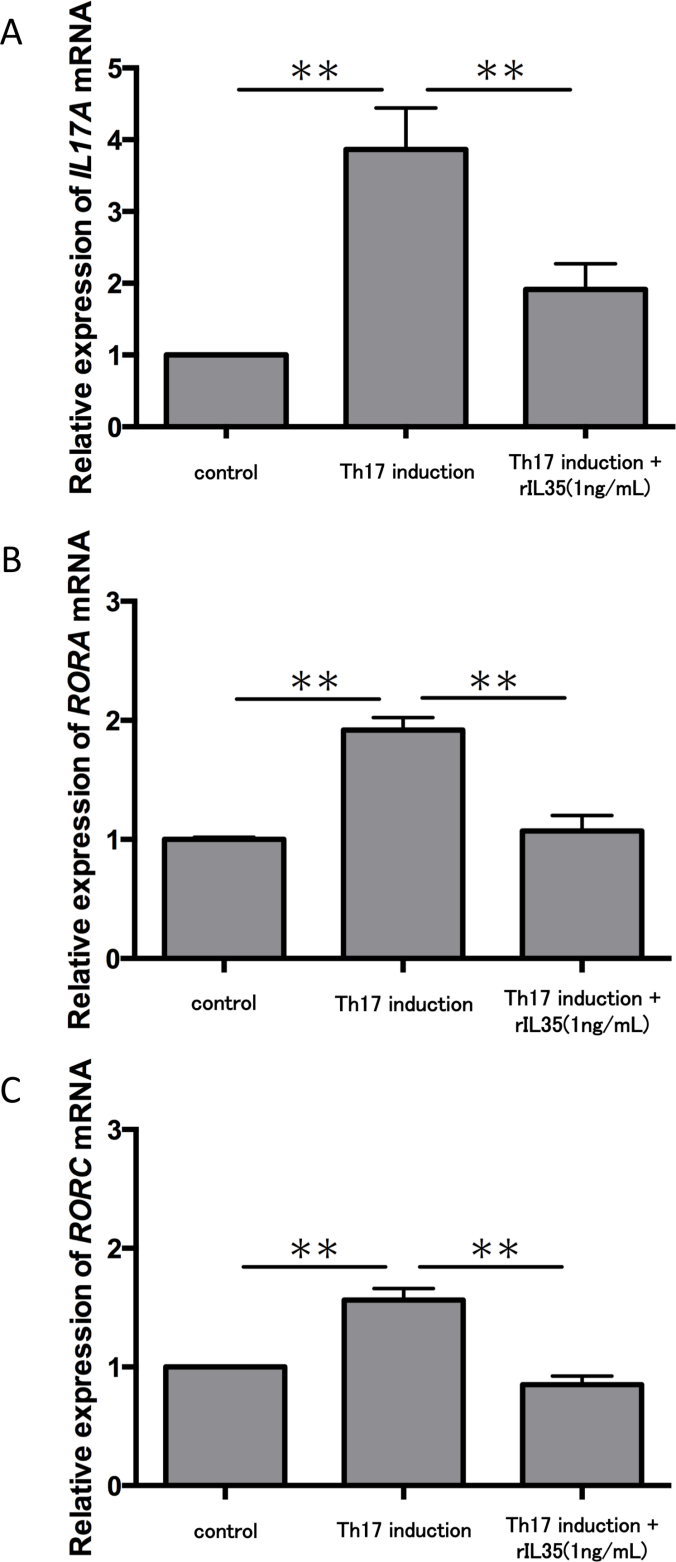
Effect of IL-35 on expression of *IL17A*, *RORA* and *RORC* mRNA. After Th17 cell differentiation by cytokine cocktail, cells were cultured with or without rIL-35 (1 ng/mL) for 2 h. *IL17A* (A), *RORA* (B) and *RORC* (C) mRNA expression was determined and normalized to *ACTB*. Values are shown as the mean ± standard error of three independent experiments. ^∗∗^*P* < 0.01, one-way repeated measures ANOVA followed by the non-parametric Bonferroni multiple comparisons test.

We next tested the expression of *IL17A* mRNA in cytokine-induced Th17 cells with or without rIL-35 (Fig. 3A). The expression of *IL17A* mRNA was significantly increased in the polarized Th17 cells (*P* < 0.01), and the induction was significantly inhibited by addition of rIL-35 (*P* < 0.01). We also examined the mRNA expression of *RORA* (Fig. 3B) and *RORC* (Fig. 3C). We confirmed a significant decrease of *RORA* and *RORC* mRNA expression by addition of rIL-35 (*P* < 0.01) (Figs. 3B and 3C). Thus, IL-35 might inhibit *IL17A* mRNA expression by inhibition of *RORA* and *RORC* mRNA expression.

**Figure 4:**
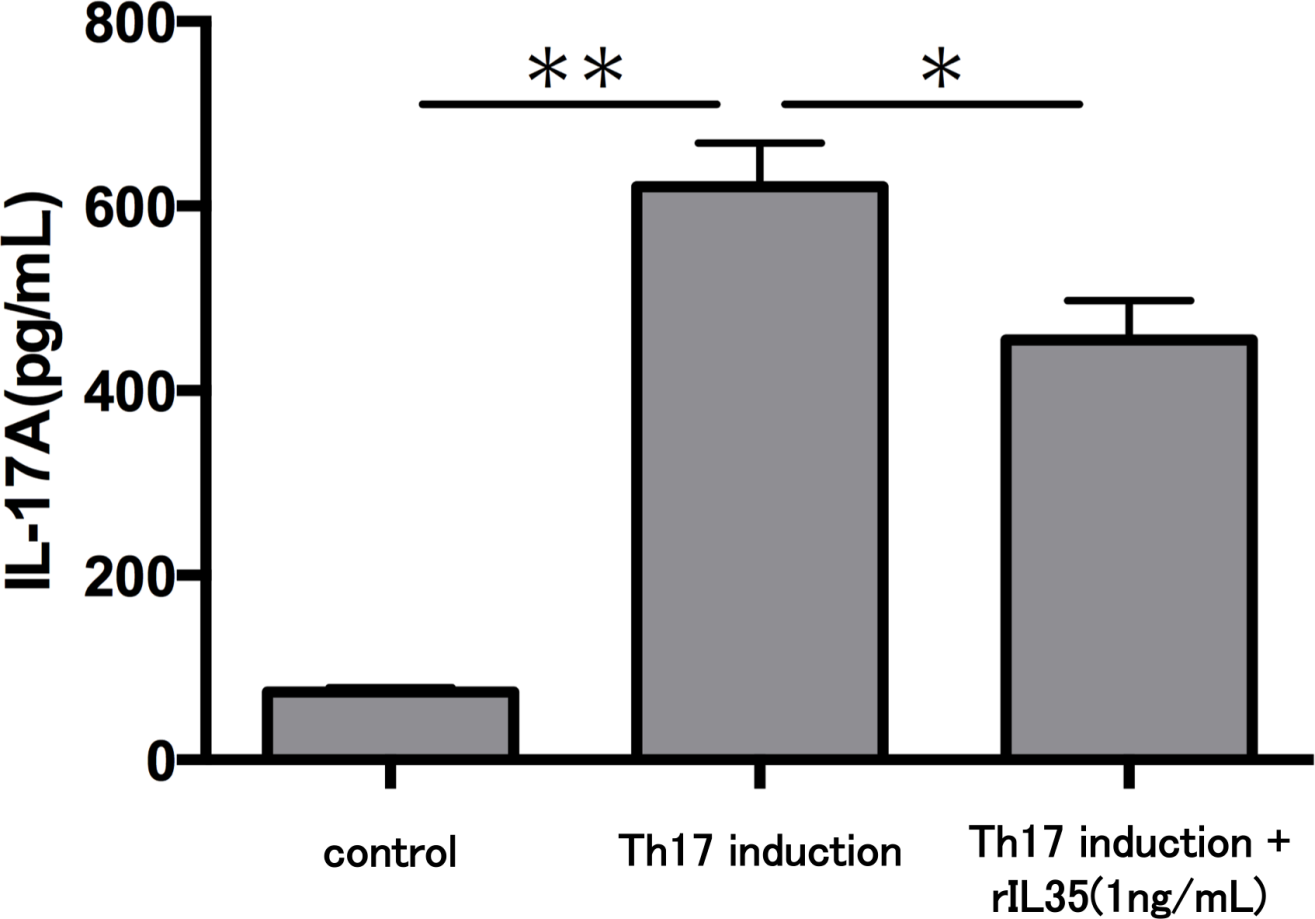
Effect of IL-35 on IL-17A production in Th17 cell. After Th17 cell differentiation by cytokine cocktail, cells were cultured with or without rIL-35 (1 ng/mL) for 24 h and IL-17A production in the culture supernatant was evaluated. Values are shown as the mean ± standard error of three independent experiments. ^∗^*P* < 0.05 and ^∗∗^*P* < 0.01, one-way repeated measures ANOVA followed by the non-parametric Bonferroni multiple comparisons test.

Finally, we investigated the levels of IL-17A production in Th17 cells with or without rIL-35 (Fig. 4). We found significantly decreased levels of IL-17A production in the rIL-35-treated groups compared with untreated Th17 cells (*P* < 0.05).

## Discussion

In this study, we found a direct inhibitory effect of IL-35 on IL-17 production in Th17 cells. IL-35 inhibited the expression of *RORA* and *RORC* mRNA during Th17 cell differentiation. These results suggest that IL-35 might inhibit ROR*α* and ROR*γ* t in Th17 cells. To the best of our knowledge, this is the first report on the direct effect of IL-35 on Th17 cells.

Previous studies have reported that Th17 cells in human periodontal tissue were increased in CP patients compared with healthy controls (Adibrad et al., 2012; Cardoso et al., 2009). Indeed, another group reported that the quantity of Th17 cells in peripheral blood from CP patients was significantly decreased after non-surgical periodontal therapy (Zhao et al., 2011). Another study showed that the levels of several inflammatory cytokines were significantly increased in serum and gingival tissue from CP patients compared with healthy controls (Gorska et al., 2003). However, data on an increase of Th17 cells in peripheral blood from patients with periodontitis remain inconclusive. In this study, there were no significant differences in any T cell populations between healthy controls and CP patients, although the percentage of IL-17A^+^CD4^+^ T cells in CP patients was slightly elevated (1.7 times) compared with that in healthy participants. A larger sample size might provide further insights, which will be performed in future research.

In this study, we found that IL-35 directly inhibited IL-17 production in Th17 cells. Several studies have reported that IL-35 inhibited Th17 cell differentiation and IL-17 production (Vignali & Kuchroo, 2012; Zhao et al., 2011). Further, it has been proposed that IL-35 suppresses Th17 cell differentiation by stimulating IL-10-producing CD4^+^ T cells (Kochetkova et al., 2010). Our previous results showed that IL-35 was significantly higher in gingival crevicular fluid (GCF) and gingival tissue from CP patients than those of healthy participants (Mitani et al., 2015). Another group also demonstrated that IL-35 in GCF and serum of CP patients were significantly higher than the control group (Liu, Jin & Lin, 2015). Based on these results, we speculate that IL-35 might play important roles in the pathogenesis of periodontitis via suppression of Th17 cells. However, the direct inhibitory effect of IL-35 on Th17 cell induction and IL-17 production is not completely understood. However, Parachuru et al. (2014) reported that the presence of IL-17^+^ cells in periodontal tissue was very low. This implies that the role of Th17 cells might be limited in periodontitis. Thus, careful consideration of the role of IL-35 in periodontitis will be necessary in the future.

Moreover, this is the first report to demonstrate that a part of this mechanism might be due to suppression of ROR*α* and ROR *γ*t . ROR*α* and ROR *γ*t are known as the Th17-specific transcription factors. Yang et al. reported that overexpression of ROR*α* promoted Th17 cell differentiation and IL-17 expression in mice. Furthermore, the authors also suggested that ROR*α* exerts a synergistic effect with ROR*γ*t in Th17 cell differentiation (Yang et al., 2008). Although Du et al. (2008) reported that Foxp3, a key transcription factor in the development and function of Treg cells, also inhibits ROR*α*-specific transcriptional activation in humans. The mechanisms leading to differentiation of Th17 cells have been well established in mice, they are still poorly understood in humans. The present results also suggest that IL-35 may suppress *RORA, RORC* and *IL17A* mRNA and IL-17 production through a direct effect on Th17 cells.

### Conclusions

The present study suggests that IL-35 could directly suppress IL-17 expression via ROR*α* and ROR*γ*t inhibition to restrain the excessive immune response in inflammatory conditions such as periodontitis. Further studies are required to confirm this hypothesis.

##  Supplemental Information

10.7717/peerj.2999/supp-1 Raw DataS1

## Additional Information and Declarations

### Data Availability

The following information was supplied regarding data availability: The raw data has been supplied as a Supplementary File.
